# Hepatoprotective Effects of (−) Epicatechin in CCl_4_-Induced Toxicity Model Are Mediated via Modulation of Oxidative Stress Markers in Rats

**DOI:** 10.1155/2021/4655150

**Published:** 2021-12-22

**Authors:** Khadijah B. Alkinani, Ehab M. M. Ali, Turki M. Al-Shaikh, Jalaluddin A. Awlia Khan, Tahani M. Al-naomasi, Soad S. Ali, Asaad A. Abduljawad, Osama F. Mosa, Tariq A. Zafar

**Affiliations:** ^1^Public Health Department, Health Sciences College at Leith, Umm Al Qura University, Makkah, Saudi Arabia; ^2^Department of Biochemistry, Faculty of Science, King Abdulaziz University, Jeddah, Saudi Arabia; ^3^Division of Biochemistry, Chemistry Department, Faculty of Science, Tanta University, 31527 Tanta, Egypt; ^4^Department of Biology, College of Science and Arts at Khulis, University of Jeddah, Jeddah, Saudi Arabia; ^5^Chemistry Department, Faculty of Science, Hail University, Hail, Saudi Arabia; ^6^Faculty of Medicine, Assiut University, Asyut, Egypt; ^7^Yousef Abdul Latif Jameel Chair of Prophetic Medicine Application, King Abdulaziz University, Jeddah, Saudi Arabia; ^8^Biochemistry Department, Bukhara State Medical Institute Named after Abu Ali Ibn Sino, Bukhara, Uzbekistan

## Abstract

**Objective:**

(−) Epicatechin (EP) is a naturally occurring antioxidant flavonoid found in some green plants. The current study was designed to evaluate the potential role of antioxidant mechanisms in the hepatoprotective properties of EP using the carbon tetrachloride (CCl_4_)-induced acute liver injury model.

**Materials and Methods:**

Rats (*n* = 7 per group) were divided into five groups including control group, (−) epicatechin group (20 mg·kg^−1^ body weight), CCl_4_ group (1 mL^−1^ body weight), CCl_4_-EP treatment group, and CCl_4_-silymarin (SILY) group. The levels of enzymes including hepatic malondialdehyde (MDA), glutathione (GSH), catalase (CAT), glutathione S-transferase (GST), nitric oxide synthase (NOS), glutathione peroxidase (GPx), and cytochrome P450 (CYP450) were analyzed via enzyme-linked immunosorbent assay (ELISA). Histological studies were performed on all groups to assess the regenerative effects of test sample and compare it with the control group.

**Results:**

Test compound EP and standard drug silymarin (SILY) considerably reduced liver function enzyme levels in the blood, which were raised by CCl_4_ administration, and increased serum albumin and total protein (TP) concentrations. The hepatic malondialdehyde (MDA) level was considerably declined, whereas glutathione (GSH), catalase (CAT), glutathione S-transferase (GST), nitric oxide synthase (NOS), glutathione peroxidase (GPx), and cytochrome P450 (CYP450) levels were upregulated in the EC-treated groups. The hepatoprotective results of the study were further confirmed via the histological assessments, which indicated a regeneration of the damaged hepatic tissue in treated rats.

**Conclusions:**

The results of this study revealed a significant protective efficacy of EP against CCl_4_-induced liver injury, which was potentially mediated via upregulation of antioxidant enzymes and direct scavenging effects of the compound against free radicals.

## 1. Introduction

The liver plays a key role in metabolism, storage, and secretion and in the detoxification of harmful chemicals [1]. Carbohydrates, proteins, and fats are mainly metabolized in the liver [2]. The liver function can be negatively affected by oxidative stress resulting from exposure to various xenobiotics (naturally occurring harmful compounds such as free radicals and hydroperoxides) [3, 4]. The liver can also be damaged by certain chemicals including paracetamol, CCl_4_, and polycyclic aromatic hydrocarbons. Hepatic injury caused by chronic exposure to these chemicals and via infectious agents may lead to progressive liver fibrosis with ultimate cirrhosis and liver failure [3]. Hepatic diseases represent the fifth most prevalent cause of death according to a statistical study done in the United Kingdom [5, 6]. In 2010, the “New Global Burden of Disease” estimated more than one million deaths due to liver cirrhosis [7]. Although liver issues can be life-threatening and are known to have a high mortality rate, there is no complete treatment or prevention. Alternatively, herbal products and pure compounds are under consideration for the preventive and therapeutic outcomes in liver diseases [8].

(−) Epicatechin (EP), an antioxidant flavonoid, belongs to the catechin group and has numerous health benefits [9]. It is found in fruits, vegetables, and beverages. It can also be obtained from green tea, tannins, grape seeds, and strawberries in very high amounts [10]. The previous studies revealed that the protective effects of EP are mediated through oxidizing and reducing glutathione, protecting tissues from any oxidative stress [11]. The studies have also shown that flavonoid-rich diets can reduce the risk of oxidative stress caused by excessive free radicals [3, 9]. The effect of prostacyclin (also known as prostaglandin I2 or PGI2) in relation to CCl_4_-induced liver injury was previously reported [8] and found that after in vitro poisoning with CCl_4_, amino acid incorporation could be partially restored by PGI2. PGI2 can reduce accumulated triglyceride and serum glutamic oxaloacetic transaminase also known as SGOT in the liver. Importantly, it can also increase liver glycogen content 24 hours after CCl_4_-induced injury [12]. In 2002, a study demonstrated the efficacy of EP in rat liver epithelial cells. It was noted at the end of the study that the transport of connexin 43 between cellular compartments was affected by tetradecanoylphorbol acetate (TPA), and it was also noted that this effect could be neutralized by EP or genistein [13]. Cui and his colleagues in 2014 conducted a study on liver protection with an extract rich in polyphenols (HMTP) against carbon tetrachloride-induced liver injury in mice. The protection of HMTP (extracted from Huangshan Maofeng) against changes in the liver caused by CCl_4_ was confirmed after a pathological study. As a result, it has been suggested that hepatoprotection can be achieved from extracts containing polyphenols [14]. In 2019, a study proved that hepatic veno-occlusive disease (VOD) can be reduced by EP by stopping hepatic oxidation through nuclear factor erythroid 2-related factor 2 (Nrf2). EP may prevent hepatitis by terminating the signaling pathway nuclear factor-kappa B (NF-*κ*B) activated by protein 60 [15]. Another study determined the efficacy of EP protection in rats concerning the alteration of oxidative stress factors and inflammation following the treatment with cypermethrin (CYP). Furthermore, the levels of pro-inflammatory cytokines, nucleic acid marker, interleukin 6 (IL-6), nitroguanidine, and tumor necrosis factor-alpha (TNF-*α*) were increased due to CYP. EP administration reduced the pro-inflammatory and antioxidative effects in rats exposed to CYP [16].

Based on the protective potentials of EP, this study was designed to evaluate its hepatoprotective potentials in CCl_4_-induced toxicity models. The effect of EC on liver enzymes, MDA level, reduced glutathione (GSH), glutathione peroxidase activity (GPx), catalase activity (CAT), nitric oxide synthase (NOS), glutathione S-transferase (GST), and cytochrome P450 was evaluated. Further, the histological and histopathological examinations were performed for various treated groups to validate the protective and regenerative effects of EC.

## 2. Materials and Methods

### 2.1. Animals

Adult male Wistar rats weighing 238.5 ± 38.5 g were used in this study. The animals were purchased from the Central Animal House, King Fahd Medical Research Center at KAU, Jeddah, KSA. The rats were fed standard rat pellets and water *ad libitum*. The protocol for animal care and handling used in this study was approved by the Animal Care and Use Committee, KAU, with Reference No: 520-18.

### 2.2. Chemicals and Drugs

Silymarin (SILY) and EP were purchased from Sigma-Aldrich (St. Louis, Missouri, USA; MDL numbers MFCD00075648 and MFCD01776359), respectively. Carbon tetrachloride (CCl_4_) was purchased from the Laboratory of Faculty of Sciences, KAU. Dimethyl sulfoxide (DMSO) and other consumables were purchased from authentic distributors. (−) Epicatechin was dispersed in DMSO and distilled water, whereas silymarin solutions were prepared in distilled water.

### 2.3. Experimental Design

The rats were randomly divided into five groups with each group containing seven animals (*n* = 7) as follows. The following groups were devised for the study:

  Group I (control/placebo group): the animals of this group were maintained on normal diet and were used as the placebo control group.
  Group II (EC group): the animals received (−) epicatechin orally (20 mg·kg^−1^ body weight for 3 weeks) [11].
  Group III (CCl_4_ group): this is the disease control group. The animals of this group received CCl_4_ (mixed with olive oil 50%) [17] intraperitoneally (1 ml·kg^−1^ body weight two times/week continued for 3 weeks).
  Group IV (CCl_4_ and EP group): the animals of this group received CCl_4_ intraperitoneally (1 ml·kg^−1^ body weight twice/week continued for 3 weeks) and (−) epicatechin orally (20 mg·kg^−1^ body weight for 3 weeks).
  Group V (CCl_4_ and SILY group): the animals of this group received CCl_4_ intraperitoneally (1 ml·kg^−1^ body weight twice/week for 3 weeks) and standard hepatoprotective drug silymarin orally (50 mg·kg^−1^ body weight of for 21 days) [18].

The rats were fasted for 12 hours, euthanized, and blood samples were collected. Blood was set for coagulation at the room temperature. After that, centrifugation was done at 2500 rpm for 15 minutes to separate the serum. For the biochemical assessment of liver function, the serum was kept at −20°C. The animals' livers were dissected and cut into small pieces; some pieces were immediately frozen under −80°C for ELISA examination and antioxidant enzyme detection, and other pieces were fixed in 10% neutral buffered formalin for histopathological studies.

### 2.4. Liver Function Assessment

Alanine amino transaminase (ALT), aspartate amino transaminase (AST), and alkaline phosphatase (ALP) [19] were measured by an automated analyzer (FLEXOR EL200, France), and several different liver markers were measured, including total protein (TP) and albumin following previously reported standard procedures [20].

### 2.5. Biochemical Analysis of Antioxidant Enzymes

The liver sections were homogenized in 50 mM K_3_PO_4_ at pH of 7.5 and 1 mM EDTA for the measurements of CAT, NO, GPx, and GSH. Sonication was performed twice on homogenized tissues with interval of 30 s at 4°C. After that, centrifugation was applied for 10 minutes at 4000 rpm·min^−1^. The concentrations of the enzymes were estimated following previously reported protocols [20–22].

### 2.6. Measurement of Malondialdehyde (MDA) Level

The determination of MDA level in the hepatic homogenates was performed using kits from Biodiagnostic, Egypt. The adducts were produced when thiobarbituric acid reacted with homogenate in a water bath and were separated by *n*-butanol. Malondialdehyde was calculated by the difference in optical densities (ODs), which were produced at different wavelengths of 535 nm and 525 nm. The results were expressed as nmol·g^−1^ tissue [23].

### 2.7. Reduced Glutathione (GSH) Analysis

GSH is the most important antioxidant synthesized in cells. It is a reducing molecule, which can react with oxygen species by neutralizing the unpaired electrons that make them highly reactive and dangerous. GSH level in liver cell homogenate was determined following previously reported standard protocol [24] using biodiagnostic assay kits. Briefly, glutathione was added into GSH monoclonal precoated wells and then incubated. Biotin-labeled anti-GSH antibodies were added to combine with HRP-conjugated streptavidin, which forms an antigen-antibody complex. After incubation, the enzymes that remained unbound were washed and removed. Substrates A and B were added to the solution, and the change in color was observed. There is a positive correlation between the concentration of rat GSH and the shades of the solution. The concentration of GSH was expressed as mmol g^−1^ tissue.

### 2.8. Estimation of Glutathione Peroxidase Activity (GPx)

Kits provided by Biodiagnostic, Egypt, were used to determine the GPx of the liver homogenates. This was calculated in a coupled enzyme assay with glutathione reductase by calculating the oxidation of nicotinamide adenine dinucleotide phosphate keeping hydrogen peroxide (H_2_O_2_) as the substrate at 340 nm [25]. It was demonstrated in nmol/min/mg protein.

### 2.9. Estimation of Tissue Catalase Activity (CAT)

The determination of CAT in the hepatic homogenates was performed using kits from Biodiagnostic, Egypt, and was calculated according to Aebi [26]. Hydrogen peroxide reacts with CAT enzyme of known amount. The reaction was stopped after the addition of catalase inhibitor after exactly one minute. The rest of the hydrogen peroxide forms a chromophore by reacting with DHBS and AAP. This chromophore has a color intensity inversely related to the quantity of CAT present in the original sample. The absorbance of samples was observed at 510 nm. It was demonstrated in *µ*mol/min/mg [23].

### 2.10. Analysis of Nitric Oxide Synthase (NOS)

The level of NOS in homogenized hepatic tissues was measured using specific enzyme-linked immunosorbent assay (ELISA) kits, provided by Bioassay Technology. Nitric oxide synthase in liver cell lysates was calculated by the procedure reported previously [24]. NOS was added to the precoated NOS monoclonal antibody wells and then incubated. Anti-NOS biotin-labeled antibodies combined with streptavidin-HRP were added, forming an immune complex. The enzymes remained unbound after the incubation was removed and washed. Two substrates A and B were added, which turned the color of the solution into blue, which then changed into yellow due to the acid. There is a positive correlation between the solution shades and the concentration of NOS [27]. The concentration of NO was expressed as pg·mg^−1^ protein.

### 2.11. Determination of Glutathione S-Transferases (GST)

The level of GST in homogenized liver tissues was measured using ELISA kits. Kits were provided by Biodiagnostic, Egypt, and GST was calculated via previously reported procedure [28]. The procedure relied upon the conjugation of GSH with CDNB. This occurs when GST forms adduct of 2,4-dinitrophenyl-S-glutathione. This adduct was calculated via a beam spectrophotometer at 340 nm.

### 2.12. Assessments of Cytochrome P450

Cytochrome P450 in hepatic homogenates was determined utilizing kits given by Bioassay Technology. For the analysis of the cytochrome P450 1A2 (CYP1A2), these kits use biotin double-antibody sandwich technology-based ELISA. CYP1A2 in cell lysates was calculated by the procedure given by [24]. CYP1A2 was added to the CYP1A2 precoated monoclonal antibody wells, which were later set for incubation. After the incubation of these cells, the immune complex was formed by the addition of anti-CYP1A2 biotin-labeled antibodies and was combined with streptavidin-HRP. Those enzymes that were not bound were removed and washed. Then, we added two substrates A and B. As a result, the color of the solution changed to blue, which then changed to yellow. There is a positive correlation between the concentration of (CYP1A2) and the shades of the solution.

### 2.13. Histological and Histopathological Studies

The rats were dissected via an abdominal incision after anesthetization. The livers of the rats were extracted for microscopic histopathological examinations. The liver was cut into slices, fixed in 10% formalin, washed, dehydrated in the ascending graded series of alcohol cleared in xylene, and embedded in paraffin. The sections of 5 *μ*m thickness were stained with hematoxylin and eosin (H&E), and other sections were stained with Masson's trichrome. These sections were examined under the light microscope (Olympus BX61, USA) with a digital camera (Olympus DP72, USA). The photographs with different magnifications were then screened to study the liver injury and the protection efficiency.

### 2.14. Statistical Study

All results were expressed as mean ± SD. The data were analyzed utilizing GraphPad Prism 3.0 Software. The differences among the experimental groups were detected by *t*-test. The values of *p* ≤ 0.05 were considered statistically significant.

## 3. Results

### 3.1. (−) Epicatechin Positively Modulates Liver Function Parameters

The concentrations of ALT, AST, ALP, TP, and albumin were measured in samples to assess liver function. It was observed that the activity of ALT, AST, and ALP was significantly increased (*p* < 0.001), while TP and serum albumin levels were noticeably decreased (*p* < 0.001) in CCl_4_-injected rats compared with the control group. There was a decrease in AST, ALP, and ALT levels in group IV (CCl_4_ and EP) and group V (CCl_4_ and SILY), whereas TP and albumin levels were increased when compared with group III (CCl_4_) (*p* < 0.001). Furthermore, the level of AST, ALP, and ALT was decreased in the SILY and CCl_4_ groups compared with group I (*p* < 0.01, 0.01, and 0.05). The deficiency was improved by EP treatment (*p* < 0.001) and SILY treatment (*p* < 0.01) (Table 1).

### 3.2. (−) Epicatechin Modulates MDA and GSH in the CCl_4_ Toxicity Model

**Figure 1:**
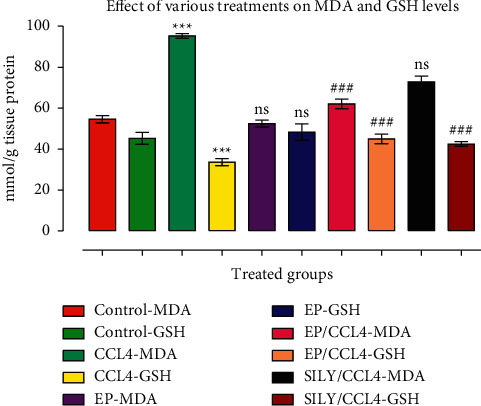
(−) Epicatechin and silymarin's effect on liver MDA and GSH activities caused by carbon tetrachloride. The data are presented as the mean ± SD (*n* = 7). ns: values not significantly different when compared with the CCl_4_-treated group. ^*∗∗*^*p* < 0.01 and ^*∗∗∗*^*p* < 0.001 vs. CCl_4_ group (GSH group); ^##^*p* < 0.01 and ^###^*p* < 0.001 values vs. CCl_4_ group (MDA group).

The results of the oxidative stress changes are summarized in Figure 1. An increase in the level of MDA was observed after CCl_4_ administration compared with the control group (*p* < 0.001). The animals in group V (CCl_4_ and SILY) also showed an increase in MDA in comparison with the control group (*p* < 0.01) (Figure 1). The rats with CCl_4_-induced hepatic injury and treated with EP showed a decrease in MDA level compared with group III (CCl_4_) (*p* < 0.001). Similar results were observed in group V when compared with group III (*p* < 0.01). The injection of CCl_4_ decreased the liver GSH level in group III compared with the control group (*p* < 0.001). The treatment with EP and SILY in groups IV and V increased the level of GSH compared with that in group III (Figure 1).

### 3.3. Effect of (−) Epicatechin on Liver GPx and CAT Caused by CCl_4_

**Figure 2:**
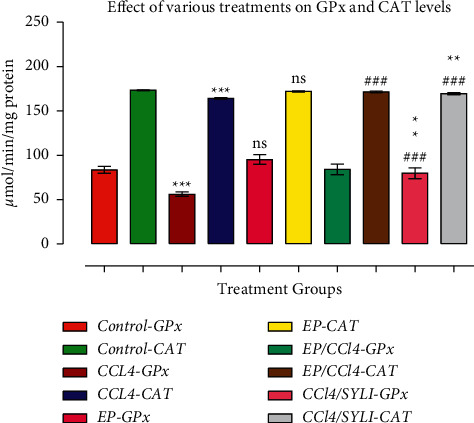
(−) Epicatechin and silymarin's effect on liver GPx and CAT activities caused by carbon tetrachloride. The data are presented as the mean ± SD (*n* = 7). ns: values not significantly different when compared with the CCl_4_-treated group. ^*∗∗*^*p* < 0.01 and ^*∗∗∗*^*p* < 0.001 vs. CCl_4_ group (GPx group); ^###^*p* < 0.001 values vs. CCl_4_ group (CAT group) and ^‡^*p* < 0.05 vs. SILY group.

The activities of GPx and CAT decreased in group III (CCl_4_) compared with the control group (*p* < 0.001). The decrease in CAT level was also noted in group V (CCl_4_ and SILY) compared with the control group (*p* < 0.01). The rats in groups IV and V showed an increased level of GPx (*p* < 0.001) and (*p* < 0.001), respectively, compared with the CCl_4_ group (Figure 2). The treatment of CCl_4_-injected rats with either EP or SILY increased CAT activity in the liver (*p* < 0.001 and *p* < 0.001, respectively) compared with the CCl_4_ group (Figure 2).

### 3.4. (−) Epicatechin and Silymarin's Effects on Liver NOS Activities

**Figure 3:**
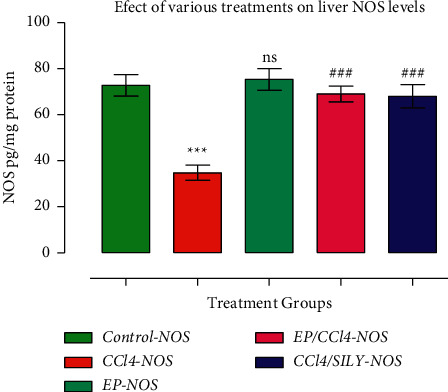
(−) Epicatechin and silymarin's effect on liver NOS activities caused by carbon tetrachloride. The data are presented as the mean ± SD (*n* = 7). ^*∗∗∗*^*p* < 0.001 vs. control group and ^###^*p* < 0.001 values vs. CCl_4_ group.

In the NOS study, it was found that the NOS level in group III (34.8 ± 10.37) was significantly reduced when compared to the control group (72.77 ± 14.88) (*p* < 0.001). The treatment of CCl_4_-injected rats with either EP or SILY increased NOS activity at *p* < 0.001 (Figure 3).

### 3.5. Effect of (−) Epicatechin on Liver GST and CYP450

**Figure 4:**
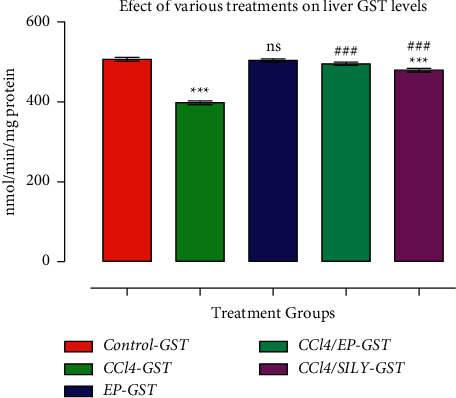
(−) Epicatechin and silymarin's effect on liver GST activity caused. The data are presented as the mean ± SD (*n* = 7). ^*∗∗∗*^*p* < 0.001 vs. control group; ^###^*p* < 0.001 values vs. CCl_4_ group; ^‡^*p* < 0.05 vs. SILY group.

**Figure 5:**
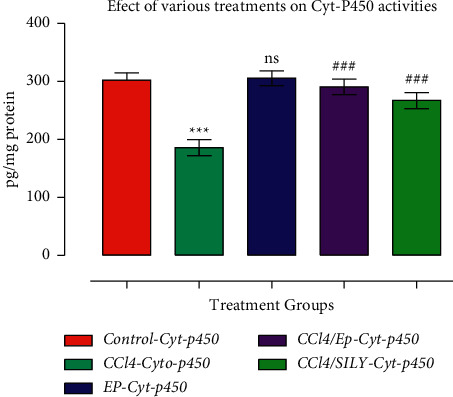
(−) Epicatechin and silymarin's effect on liver CYP450. The data are presented as the mean ± SD (*n* = 7). ^*∗∗∗*^*p* < 0.001 vs. control group; ^###^*p* < 0.001 values vs. CCl_4_ group.

**Figure 6:**
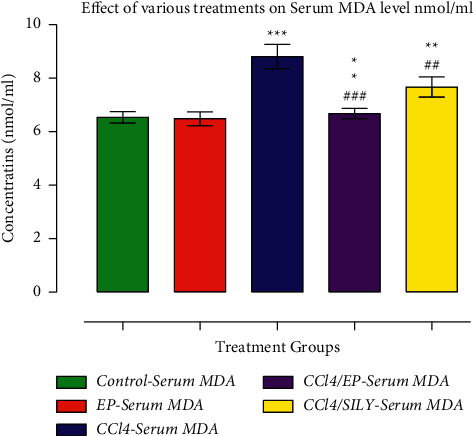
(−) Epicatechin and silymarin's effect on serum MDA. The data are presented as the mean ± SD (*n* = 7). ^*∗∗∗*^*p* < 0.001 vs. control group; ^###^*p* < 0.001 values vs. CCl_4_ group.

**Figure 7:**
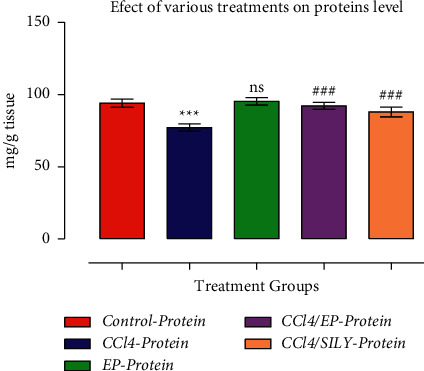
(−) Epicatechin and silymarin's effect on proteins. The data are presented as the mean ± SD (*n* = 7). ^*∗∗∗*^*p* < 0.001 vs. control group; ^###^*p* < 0.001 values vs. CCl_4_ group.

As shown in Figures 4 and 5, GST and CYP450 activities were decreased (*p* < 0.001) in group III (CCl_4_) compared with the control group. SILY treatment resulted in a significant increase (*p* < 0.001) in GST activity compared with the control group. The decline in GST and CYP450 levels after CCl3 treatment was significantly (*p* < 0.001) reversed by EP treatment. Similar results were observed in the SILY-treated groups, whereby GST and CYP450 were significantly improved (*p* < 0.001 and *p* < 0.001, respectively) when compared with group III (CCl_4_). Moreover, the increase in the GST activity of EP-treated group (group IV) was very comparable with the SILY-treated group (group V). The MDA level was significantly reduced, whereas proteins were increased as shown in Figures 6 and 7.

### 3.6. Effect of EP and SILY Treatment on Histopathological Changes in the Liver Induced by CCl_4_

To evaluate the effect of EP or SILY in the liver of CCl_4_-injected rats, we performed hematoxylin and eosin (H&E) and Masson's trichrome staining.

#### 3.6.1. Hematoxylin and Eosin (H&E) Stain

**Figure 8:**
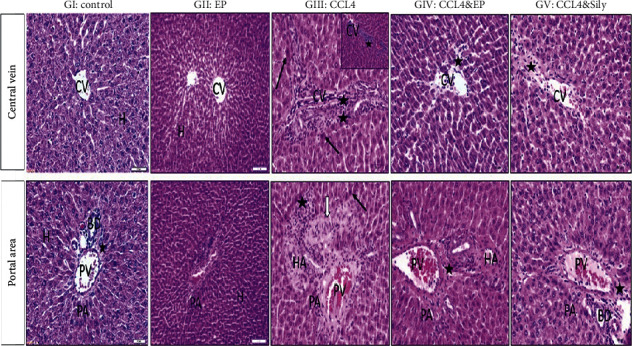
(−) Epicatechin and silymarin's effect on the liver's histopathological changes in both central vein (CV) and portal area (PA) regions caused by carbon tetrachloride. GI: control normal hepatocytes (H) in both central vein (CV) and portal area (PA) with few connective tissue cells (asterisk). BD: bile duct. GII: EP showing the normal cytoarchitecture of the lobule. The central vein is surrounded by hepatic cells separated by blood sinusoids. GIII: CCl_4_ showing cell necrosis around CV with inflammatory cells and fibroblasts (black asterisks), vascular wall (HA) thickening (white arrow) with inflammatory cells (black asterisks), and degenerating hepatocytes (black arrow) in the portal area. GIV: CCl_4_ and EP showing only a few fibrotic and inflammatory cells (black asterisks) around CV and thickening of artery (HA). Absence of fibrotic and inflammatory cells in PA (asterisks). GV: CCl_4_ and SILY. Absence of fibrotic and inflammatory cells except for a small area (asterisks) around CV and PA.

The sections from groups I and II showed a normal histological structure of hepatic tissue (Figure 8), while liver sections from the CCl_4_-treated group showed narrowing of the sinusoidal lumen and irregularity in the liver parenchyma. A large number of inflammatory cells surrounded the central vein (CV), which replaced the degenerated necrotic cells. Inflammatory cells were also present in the portal area (PA), which showed thickening of blood vessel walls. After treatment with either EP or SILY, the hepatic tissue retained its normal architecture. The severity of hepatic fibrosis decreased except for some hepatocytes that showed necrosis around the CV. These results demonstrated that EP and SILY have a vital role in improving liver fibrosis.

#### 3.6.2. Masson's Trichrome

**Figure 9:**
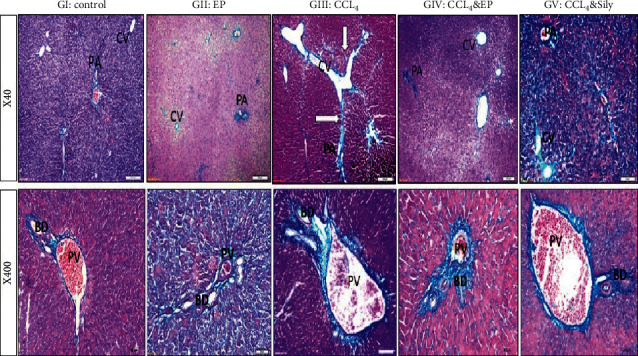
Effect of (−) epicatechin and silymarin on histopathological changes in CCl_4_-induced toxicity study. The collagen components are stained blue, and the cytoplasm appears in varying degrees of red. GI: showing few amounts of collagen fibers around the portal area (PA), portal vein (PV), and bile duct (BD). GII: no change in collagen density. GIII: collagen is increased in the portal region forming bridges between the lobules (white arrows). High-power X400 showed dilated portal vein (PV) and marked fibrosis around bile duct (BD). GIV: moderate deposition in collagen in CV, PA, and PV areas. GV: mild collagen deposition.

Collagen deposition and hepatic fibrosis were detected by Masson's trichrome stain. Collagen appeared distributed in few amounts around the central vein and portal area in the control and EP groups (Figure 9). The deposition of blue collagen and red fibers increased in the CCl_4_-treated group in CV and PA by a somewhat greater amount. After treatment with EP and SILY, the amount of collagen decreased compared with group III.

## 4. Discussion

Hepatic injury may lead to inflammation, fibrosis, and necrosis causing liver failure [29]. Phytochemicals from several plants have been used for medicinal purposes in many regions of the world [30–33]. Silymarin, a highly potent phytochemical, is used against hepatic diseases [34]. Since EP belongs to polyphenols, which are potent antioxidants against ROS-induced oxidative stress, it is used to control liver diseases [35]. CCl_4_ is commonly used in hepatotoxicity studies using experimental animal models because it causes lipid peroxidation due to the production of free radicals [36]. CCl_4_ is the best animal model characterized by free radical-induced hepatotoxicity by xenobiotics [37]. In this analysis, CCl_4_ caused significant hepatic damage and oxidative stress in animals as evidenced by altered liver function tests and antioxidant enzymes [36, 38].

This study revealed the attenuating effects of EP and SILY against CCl_4_-induced liver injury in rats. As indicated by our findings, EP administration significantly improved liver functions by decreasing blood ALT, AST, and ALP levels and increasing the levels of TP and serum albumin. EP treatment, especially in combination with SILY, declined MDA levels and ROS production, whereas NOS and CYP450 levels were upregulated. Moreover, EP and SILY have improved the hepatic histopathological changes caused by CCl_4_ administration.

This study showed that EP and SILY can protect against CCl_4_-induced liver injury. These results were in accordance with previously reported studies [39, 40], where the findings suggested that EP or SILY could be important potential protective supplements against liver injury induced by CCl_4_ in rats. It should be noted that the most common marker enzymes in the liver are AST, ALT, and ALP, whereas MDA, GSH, GPx, and CAT are vital oxidants and antioxidant balance biomarkers [41]. The toxic effects of CCl_4_ are mediated via free radicals leading to an increase in lipid peroxide, which is a major cause of CCl_4_-induced hepatic injury [42]. An increase in serum MDA was observed in this study, while the CCl_4_-treated group, in comparison with the first group, showed a significant decrease in hepatic GSH, GPx, and CAT activities. The level of MDA in the liver has been used to assess the extent of liver damage [30].

During (−) epicatechin and silymarin treatments, the level of enzymatic antioxidants showed a significant increase. This demonstrated that hepatic antioxidant activity could be maintained and restored using these compounds. Thus, oxidative damage of the liver could be treated by neutralizing it with antioxidants [43], increasing the activity of the antioxidant enzymes and inhibiting the production of MDA and lipid peroxidation [39].

GSH has an anti-hepatotoxic effect against oxidative stress [44]. H_2_O_2_ is degraded into water and oxygen by CAT, which protects hepatocytes from oxidative damage caused by H_2_O_2_ [45]. A wide range of xenobiotics and carcinogens are metabolized by the GST [46]. It has been proven from many studies that carbon tetrachloride alters GST activities [36, 47]. It was observed in this study that the level of GST was decreased in the liver of rats with CCl_4_-induced injury compared with the control group, which indicated that GST in the liver could cause tumorigenesis during chronic liver damage [38]. GST can be restored in the liver through the use of EP and SILY as they detoxify CCl_4_ and protect the liver from damage.

Oxidation of foreign chemicals occurs via cytochrome P450 enzyme [39, 48]. It also metabolizes CCl_4_ to trichloromethyl radicals leading to some harmful effects when they interact with proteins and lipids [49]. A decrease in hepatic CYP450 was also observed in this study on rats with CCl_4_-induced injury when compared to the control group. However, an increase in CYP450 activity was observed in the EP- and SILY-treated groups when compared to the CCl_4_ group. Other studies have also shown that liver cytochrome is affected by it [50]. This study showed that EP and SILY can be used to protect the liver as they have antioxidant properties. Nitric oxide synthase can also be considered as an anti-inflammatory agent [27]. NOS may play a critical role in the prevention of hepatic injury and fibrosis [51]. In the results of this work, it was observed that NOS level in the CCl_4_ group was decreased compared with the control group. Hepatic injury can be mitigated by the production of nitric oxide [17]. The treatment with EP and SILY restored the level of NOS, and reference [52] found that (−) epicatechin prevents oxidative stress and regulates nitric oxide bioavailability. Reference [53] also reported that HIF-1*α* expression was reduced with silymarin and with iNOS.

The histological study was applied to confirm the biochemical findings. Microscopic observation showed that the EP and SILY groups showed the best histopathological results compared with the CCl_4_ group. They may reduce liver fibrosis and infiltration of inflammatory cells. These protective effects against several toxins have been reported in the literature; Wang et al. demonstrated the protective activity of catechin derivative epigallocatechin gallate (EGCG) on hepatic injury caused by paracetamol. Their research showed that EGCG can reduce the occurrence of necrosis around the CV in the liver [54]. A different study indicated that catechin derivative EGCG (a beneficial plant compound called polyphenol) can improve edema, steatosis, and degeneration of the hepatocytes [55]. Cao et al. showed that green tea protects liver tissue from alcohol-induced injury by reducing lipid accumulation and preventing tissue damage due to the presence of polyphenols and their antioxidant effects [48].

It was confirmed by this study that carbon tetrachloride changes the biochemical functions of the liver through histological alteration. Necrosis of hepatocytes and their replacement with other inflammatory cells in the third group injected with carbon tetrachloride explained the fluctuation of the enzymes of the liver and other sera possibly due to the production of free radicals. The same findings were also discussed by [56] who reported that injuries caused by CCl_4_ in the liver caused inflammatory cell infiltration, fibrous bridge formation, and perivenular cell necrosis. CCl_4_ depicts its hepatotoxic effects through several pathways: dilation and congestion of blood vessels, abnormal mitosis, hemosiderin deposition, bile duct proliferation, and hepatocyte necrosis [57]. EP coupled with SILY exhibited considerable hepatoprotective effects in the liver of rats treated by CCl_4_. Among these two, EP proved to be more effective [58]. As discussed, EP is a very strong antioxidant [35] and it can be used to protect the liver from many toxins.

## 5. Conclusion

The findings of this study revealed that EP ameliorates CCl_4_-induced hepatotoxicity and oxidative stress in rats. The intraperitoneal injection of CCl_4_ increases the activity of ALT, AST, and ALP, decreases the levels of TP and serum albumin, increases the level of MDA and ROS production, and decreases the level of NOS and CYP450. Oral administration of EP and SILY mitigates all of these harmful effects in the livers of rats. They reduce oxidative stress, suppress inflammatory cell infiltration, increase the regenerative capacity of damaged tissues, and reduce liver apoptosis. Thus, we believe that the use of natural products such as EP and SILY can aid in reducing the toxic effects resulting from exposure to xenobiotics such as CCl_4_ and other various toxic substances.

## Figures and Tables

**Table 1:** Effect of (−) epicatechin on ALT, AST, ALP, total protein, and albumin concentrations.

Samples	Control	EP	CCl_4_	CCl_4_-EP	CCl_4_-SILY	Samples	Control	EP	CCl_4_	CCl_4_-EP	CCl_4_-SILY
Serum ALT activity (U·L^−1^)						Serum AST activity (U·L^−1^)					
1	15.23	9.84	80.15	19.2	13.33	1	36.31	24.12	118.09	25.07	35.54
2	11.42	11.11	41.74	10.79	18.57	2	30.28	30.45	129.83	35.87	55.22
3	14.6	10.15	37.45	12.06	13.33	3	25.07	21.9	124.12	30.15	28.24
4	13.33	14.6	26.66	18.25	19.84	4	24.75	22.85	143.16	29.83	48.72
5	12.69	8.89	55.23	17.93	21.74	5	25.07	19.67	125.39	26.34	53.17
6	10.15	8.57	66.32	12.06	25.98	6	28.24	26.34	95.07	26.97	30.46
7	11.74	13.33	72.38	18.25	30.74	7	18.72	26.97	121.9	39.67	27.29
8	9.2	9.84	82.38	22.45	35.40	8	23.17	19.67	84.12	35.8	52.99
9	10.79	12.06	27.5	18.22	19.00	9	25.07	15.87	130.15	26.7	22.21
10	7.93	8.25	73.65	17.00	25.00	10	36.87	30.15	93.17	36.2	50.34
Mean	**11.70**	**10.66**	**56.34** ^*∗∗∗*^	**16.62** ^###^	**22.29** ^*∗*###^	Mean	**27.35**	**23.79**	**116.5** ^*∗∗∗*^	**31.26** ^###^	**40.418** ^###^^,^^*∗∗*^
^*∗*^ *p* vs. control		**0.82**	**4.04**	**0.22**	**0.027**	^*∗*^ *p* vs. control		**0.14**	**8.03**	**0.53**	**0.011**
^#^ *p* vs. CCl_4_ group				**5.50**	**3.30**	^#^ *p* vs. CCl_4_				**3.14**	**6.68**
^#^ *p* vs. SILY group				**0.078**		^#^ *p* vs. SILY				**0.07**	

Serum ALP activity (U·L^−1^)						Serum albumin level (g %)					

1	58.81	49.62	165.46	64.11	111.19	1	3.064	2.74	2.78	3.12	2.59
2	102.92	49.62	168	79.95	128.76	2	3.206	3.46	2.50	2.77	2.55
3	101.01	68.92	162.66	45.03	73.52	3	3.149	3.70	2.86	2.76	3.03
4	85.46	85.46	110.54	36.76	67.08	4	2.809	3.50	2.67	3.59	3.18
5	102.44	94.65	107.52	48.70	55.95	5	3.20	3.01	2.22	3.12	3.18
6	44.70	45.03	175.35	61.57	110.28	6	3.45	3.26	2.11	3.03	3.39
7	38.57	36.76	91.46	38.59	116.71	7	3.21	3.64	2.10	3.26	3.39
8	38.59	88.46	151.46	97.23	67.30	8	3.45	2.95	2.81	3.21	3.18
9	40.11	98.00	162.49	100.3	143.47	9	3.33	3.54	2.25	3.4	3.43
10	52.95	51.46	172.60	103.2	55.35	10	2.95	3.21	2.56	3.56	2.11
Mean	**66.56**	**66.80**	**146.75** ^*∗∗∗*^	**67.54** ^###^	**92.96** ^###^^,^^*∗∗*^	Mean	**3.18**	**3.30**	**2.49** ^*∗∗∗*^	**3.18** ^###^	**3.00** ^###^
^*∗*^ *p* vs. control		**0.983**	**1.03**	**0.93**	**0.01**	^*∗*^ *p* vs. control		**0.327**	**9.90**	**0.97**	**0.27**
^#^ *p* vs. CCl_4_ group				**7.83**	**1.10**	^#^ *p* vs. CCl_4_				**4.62**	**0.00075**
^#^ *p* vs. SILY group				**0.06**		^#^ *p* vs. SILY				**0.30**	

The data are presented as the mean ± SD (*n* = 7). *p*^*∗*^ ≤ 0.05 vs. control group and *p*^#^ ≤ 0.05 vs. CCl_4_ group. Control: placebo group; EP: (−) epicatechin group; CCl_4_-EP: CCl_4_ and (−) epicatechin group; SILY: silymarin group.

## Data Availability

The data in the manuscript belong to the research work of Khadijah B. Alkinani and will be provided to researchers upon request.

## Abbreviations

EP: (−) Epicatechin
CCl_4_: Carbon tetrachloride
SILY: Silymarin
MDA: Malondialdehyde
GSH: Glutathione
CAT: Catalase
GST: Glutathione S-transferase
NOS: Nitric oxide synthase
GPx: Glutathione peroxidase
CYP450: Cytochrome P450
TPA: Tetradecanoylphorbol acetate
VOD: Veno-occlusive disease
Nrf2: Nuclear factor erythroid 2-related factor 2
NF-*κ*B: Nuclear factor-kappa B
CYP: Cypermethrin
TNF-*α*: Tumor necrosis factor-alpha
DMSO: Dimethyl sulfoxide
ALT: Alanine amino transaminase
AST: Aspartate amino transaminase
ALP: Alkaline phosphatase
TP: Total protein
ODs: Optical densities
H_2_O_2_: Hydrogen peroxide
ELISA: Enzyme-linked immunosorbent assay
CYP1A2: Cytochrome P450 1A2
H&E: Hematoxylin and Eosin.
